# Utilization of Thromboelastogram and Inflammatory Markers in the Management of Hypercoagulable State in Patients with COVID-19 Requiring ECMO Support

**DOI:** 10.1155/2021/8824531

**Published:** 2021-01-15

**Authors:** Angela Smolarz, Paul McCarthy, Aaron Shmookler, Vinay Badhwar, Awori J. Hayanga, Ankit Sakhuja

**Affiliations:** ^1^Division of Cardiovascular Critical Care, Department of Cardiovascular and Thoracic Surgery, West Virginia University, Morgantown, WV, USA; ^2^Department of Pathology, Anatomy and Laboratory Medicine, West Virginia University, Morgantown, WV, USA; ^3^Division of Cardiac Surgery, Department of Cardiovascular and Thoracic Surgery, West Virginia University, Morgantown, WV, USA; ^4^Division of Thoracic Surgery, Department of Cardiovascular and Thoracic Surgery, West Virginia University, Morgantown, WV, USA

## Abstract

The role of extracorporeal membrane oxygenation (ECMO) in the management of critically ill patients with COVID-19 is evolving. Extracorporeal support independently confers an increased predilection for thrombosis, which can be exacerbated by COVID-19-associated coagulopathy. We present the successful management of a hypercoagulable state in two patients who required venovenous ECMO for the treatment of COVID-19. This included monitoring inflammatory markers (D-dimer and fibrinogen), performing a series of therapeutic plasma exchange procedures, and administering high-intensity anticoagulation therapy and thromboelastography- (TEG-) guided antiplatelet therapy. TPE was performed to achieve goal D-dimer less than 3000 ng/mL D-dimer units (*N* ≤ 232 ng/mL D-dimer units) and goal fibrinogen less than 600 mg/dL (*N* = 200-400 mg/dL). These therapies resulted in improved TEG parameters and normalized inflammatory markers. Patients were decannulated after 37 days and 21 days, respectively. Post-ECMO duplex ultrasound of the upper and lower extremities and cannulation sites revealed a nonsignificant deep venous thrombosis at the site of femoral cannulation in patient 2 and no deep venous thrombosis in patient 1. The results of this case report show successful management of a hypercoagulable state among COVID-19 patients requiring ECMO support by utilization of inflammatory markers and TEG.

## 1. Introduction

The spread of severe acute respiratory syndrome coronavirus-2 (SARS-CoV-2) is responsible for the 2019 coronavirus disease (COVID-19) pandemic. Evidence has emerged that the SARS-CoV-2 virus elicits a proinflammatory and hypercoagulable state manifested by elevated procoagulant factor levels and elevated fibrinogen and D-dimer levels [1]. Klok et al. have demonstrated a high incidence of thrombotic complications in patients with COVID-19 who were admitted to the intensive care unit (ICU) [2]. Among critically ill patients requiring venovenous extracorporeal membrane oxygenation (VV-ECMO) support for SARS-CoV-2, Parzy et al. recently demonstrated a 100% incidence of venous thromboembolism [3]. In this report, we describe our experience in managing hypercoagulable states in the first two cases of COVID-19 that required ECMO support at our institution.

## 2. Clinical Presentation

### 2.1. Case #1

**Figure 1:**
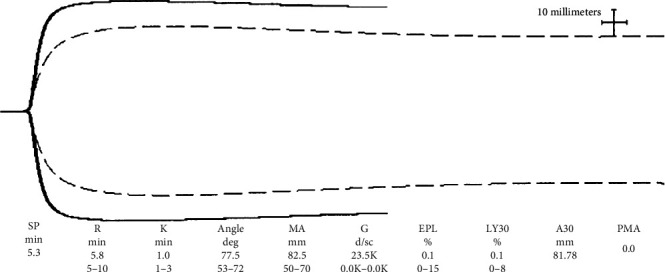
Initial TEG case #1.

Case 1 was a 41-year-old White male with morbid obesity (body mass index 50.8 kg/m^2^) who was admitted to our institution with worsening dyspnea after being diagnosed with COVID-19. His past medical history was otherwise unremarkable. He was placed on a high-flow nasal cannula and transferred to the ICU on the same day. In the ICU, he underwent conscious prone positioning and was administered inhaled nitric oxide for profound hypoxemia. His clinical condition deteriorated such that, on hospital day #3, he was intubated and mechanically ventilated; soon thereafter, he was started on VV-ECMO because of persistent and refractory hypoxemia. Prior to ECMO cannulation, ventilator settings were pressure control 20 cmH_2_O, positive end expiratory pressure (PEEP) 16 cmH_2_O, respiratory rate (RR) 14 breaths per minute, and fraction of inspired oxygen (FiO_2_) 100%. An arterial blood gas showed pH 7.26, pCO_2_ 72 mmHg, pO_2_ 79 mmHg, and bicarbonate 27 mEq/L. His initial thromboelastography (TEG) following cannulation was reflective of an underlying hypercoagulable state (Figure 1). Initial D-dimer and fibrinogen levels were 791 ng/mL D-dimer units (*N* ≤ 232 ng/mL D-dimer units) and 758 mg/dL (*N* = 200-400 mg/dL), respectively. Based on these results, he was anticoagulated with bivalirudin per institutional protocol and also underwent PlateletMapping®-guided antiplatelet therapy with aspirin. Therapeutic plasma exchange (TPE) using both albumin and plasma was performed to achieve goal D-dimer less than 3000 ng/mL and goal fibrinogen less than 600 mg/dL. The patient underwent a total of 12 1.0-1.5 volume exchange procedures. He was extubated after 5 days and decannulated from VV-ECMO after 37 days. During the patient's course, he also received 1 unit of convalescent plasma and a single dose of tocilizumab 8 mg/kg IV for cytokine release syndrome-like clinical symptoms. Bilateral lower and upper extremity duplex ultrasound performed after decannulation showed no evidence of deep vein thrombosis. The patient was discharged to a rehabilitation center 54 days after admission.

### 2.2. Case #2

**Figure 2:**
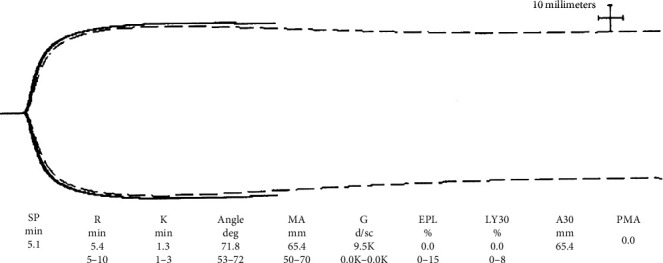
Initial TEG case #2.

Case #2 was a 55-year-old Hispanic male who was transferred from an outside facility after being intubated for COVID-19-related acute respiratory distress syndrome. His past medical history was notable for hypertension, prediabetes, and obesity (body mass index 31.2 kg/m^2^). Prior to his arrival, he received two units of convalescent plasma and tocilizumab. He was placed on VV-ECMO within hours after arriving to our facility. Prior to cannulation, ventilator settings were pressure control 14 cmH_2_O, PEEP 18 cmH_2_O, RR 20, and FiO_2_ 100%. An arterial blood gas showed pH 7.28, pCO_2_ 63 mmHg, pO_2_ 68 mmHg, and bicarbonate 26 mEq/L. His initial TEG was also consistent with a hypercoagulable state (Figure 2). Initial D-dimer and fibrinogen levels were >5000 ng/mL D-dimer units and 196 mg/dL, respectively. Antiplatelet therapy as guided by TEG was again used in addition to bivalirudin for anticoagulation. He also underwent 16 TPE procedures, replacing 1.0-1.5 volumes with a mixture of albumin and plasma, based on D-dimer and fibrinogen levels. He was extubated after 8 days and decannulated after 22 days. After decannulation, duplex ultrasound of all four extremities showed only a nonocclusive cannula-associated thrombus of the right femoral vein. The patient was discharged to a rehabilitation center 35 days after admission.

## 3. Discussion

During the early days of the pandemic, patients with COVID-19 were noted to have thrombotic complications suggesting the existence of a hypercoagulable state. Multiple studies have since confirmed this finding in that patients have developed both venous and arterial thrombotic episodes including life-threatening complications [4, 5]. Due to the availability of and experience with TEG in our ICU, we opted to utilize it to better evaluate the viscoelastic properties of these patients. Using Haemonetics TEG 5000, we observed a significant magnitude of hypercoagulability characterized by short R time, wide alpha, and large MA. Features suggestive of fibrinolytic shutdown were also noted. Fibrinolytic shutdown is defined as the presence of an elevated D-dimer and low fibrinolytic activity, which has been described as <0.8% clot lysis at 30 minutes [6]. Fibrinolytic shutdown has recently been described to be associated with increased episodes of venous thromboembolic episodes in patients with COVID-19 [7]. Both our patients had <0.8% lysis at 30 minutes on their initial TEGs. Soon after ECMO cannulation, both patients received high-intensity bivalirudin. Due to significantly elevated MA and reports of arterial thrombotic episodes in patients with COVID-19 [4], they also received aspirin, the dose for which was guided using the TEG PlateletMapping® assay. In keeping with its role in septic shock in improving the procoagulant profile, these patients were treated with a rigorous regimen of TPE [8]. Proinflammatory and procoagulant markers D-dimer and fibrinogen were used to guide TPE interventions. Patients were offered TPE for D-dimer level ≥ 3000 ng/mL D-dimer units or fibrinogen level ≥ 600 mg/dL. The first treatment was 1.5 plasma volume with subsequent treatments being 1.0 plasma volume. The proportion of fresh frozen plasma and albumin during each exchange procedure was left at the discretion of the attending physician. All TPE procedures were performed on Prismaflex using citrate as the anticoagulant of choice. With the aggressive regimen of high-intensity anticoagulation and antiplatelet therapy as guided by TEG and TPE as guided by D-dimer and fibrinogen, we were able to successfully manage hypercoagulable states in these patients while on ECMO.

Mortus et al. have recently demonstrated the utility of TEG not only to identify hypercoagulable states in COVID-19 patients but also to aid in the management [9]. ECMO use by itself is associated with over 85% risk of thrombotic events [10]. As such, patients with COVID-19 are likely at a much higher risk of thrombosis while on ECMO compared to a clotting diathesis invoked by ECMO alone. Therefore, an aggressive management strategy is necessary to prevent life-threatening thrombotic complications in COVID-19 patients on ECMO. Of the two patients in this case report, one had a partially occlusive cannula-related right femoral vein thrombus found after decannulation while on ECMO for 22 days. The other patient in this case report had no evidence of deep venous thrombi following decannulation from extracorporeal support for 37 days.

In summary, we hereby describe a strategy that combines high-intensity anticoagulation, antiplatelet therapy, and TPE—as guided explicitly by the combination of TEG, D-dimer, and fibrinogen levels—to successfully manage a SARS-CoV-2-associated hypercoagulable state in patients requiring extracorporeal support. Further studies directly exploring the benefits of this treatment strategy are needed to address the extreme burden of hypercoagulability seen in these patients.

## Data Availability

No datasets were used for this study.
